# Effect of Granulocyte Colony-Stimulating Factor on the Development of Spermatogenesis in the Adulthood of Juvenile AML Mice Model Treated with Cytarabine

**DOI:** 10.3390/ijms241512229

**Published:** 2023-07-31

**Authors:** Bara’ah Khaleel, Eitan Lunenfeld, Joseph Kapelushnik, Mahmoud Huleihel

**Affiliations:** 1 The Shraga Segal Department of Microbiology, Immunology, and Genetics, Faculty of Health Sciences, Ben-Gurion University of the Negev, Beer Sheva 8410501, Israel; khaleelb@post.bgu.ac.il; 2 The Center of Advanced Research and Education in Reproduction (CARER), Faculty of Health Sciences, Ben-Gurion University of the Negev, Beer Sheva 8410501, Israel; kapelush@bgu.ac.il; 3 Faculty of Health Sciences, Ben-Gurion University of the Negev, Beer Sheva 8410501, Israel; 4 Adelson School of Medicine, Ariel University, Ariel 4076414, Israel; eitanlun@ariel.ac.il; 5 Department of Pediatric Oncology and Hematology, Soroka Medical Center, Beer-Sheva, and Faculty of Health Sciences, Ben-Gurion University of the Negev, Beer-Sheva 8410501, Israel

**Keywords:** male infertility, spermatogenesis, acute myeloid leukemia (AML), chemotherapy, male fertility preservation, granulocyte-colony stimulating factor (GCSF), prepubertal patients

## Abstract

Pediatric acute myeloid leukemia (AML) generally occurs de novo. The treatment of AML includes cytarabine (CYT) and other medications. The granulocyte-colony stimulating factor (GCSF) is used in the clinic in cases of neutropenia after chemotherapies. We show that the administration of GCSF in combination with CYT in AML-diagnosed mice (AML+CYT+GCSF) extended the survival of mice for additional 20 days. However, including GCSF in all treatment modalities does not affect the testis’ weight or the histology of the seminiferous tubules (STs). We show that GCSF does not affect normal ST histology from AML-, CYT-, or (AML+CYT)-treated groups compared to the relevant treated group without GCSF 2, 4, and 5 weeks post-injection. However, when comparing the percentages of normal STs between the AML+CYT+GCSF-treated groups and those without GCSF, we observe an increase of 17%–42% in STs at 4 weeks and 5.5 weeks post-injection. Additionally, we show that the injection of GCSF into the normal, AML-alone, or CYT-alone groups, or in combination with AML, significantly decreases the percentage of STs with apoptotic cells compared to the relevant groups without GCSF and to the CT (untreated mice) only 2 weeks post-injection. We also show that injection of GCSF into the CT group increases the examined spermatogonial marker PLZF within 2 weeks post-injection. However, GCSF does not affect the count of meiotic cells (CREM) but decreases the post-meiotic cells (ACROSIN) within 4 weeks post-injection. Furthermore, GCSF not only extends the survival of the AML+CYT-treated group, but it also leads to the generation of sperm (1.2 ± 0.04 × 10^6^/mL) at 5.5 weeks post-injection. In addition, we demonstrate that the injection of GCSF into the CT group increases the RNA expression level of IL-10 but not IL-6 compared to CT 2 weeks post-treatment. However, the injection of GCSF into the AML-treated group reverses the expression levels of both IL-10 and IL-6 to normal levels compared to CT 2 weeks post-injection. Thus, we suggest that the addition of GCSF to the regimen of AML after CYT may assist in the development of future therapeutic strategies to preserve male fertility in AML prepubertal patients.

## 1. Introduction

Spermatogenesis is a complicated biological process that starts when the spermatogonial stem cells (SSCs) go from the self-renewal state and start to differentiate into highly specialized haploid spermatozoa [1]. The spermatogonial cells consist of different cell types that could be distinguished by the various unique protein markers. Those specific cell markers are used to identify the developmental stages of spermatogenesis, such as pre-meiotic cells that express several markers, including promyelocytic leukemia zinc finger (PLZF) [2], meiotic cells that express activator cAMP-responsive element modulators (CREMs) [3,4], and meiotic/post-meiotic cells that express ACROSIN [5]. The microenvironment surrounding the germ cells in the testis plays a key role in regulating spermatogenesis. Somatic cells (Sertoli cells, Leydig cells, and peritubular cells) and spermatogonial cells at different stages of their development secrete pro-inflammatory and immunoregulatory cytokines (IL-1α, IL-1β, IL-6, IL-1RA, and IL-10) [6], and additional growth factors/cytokines (GDNF, SCF, MCSF, and LIF) that regulate the proliferation and/or the differentiation of SSCs under normal conditions [7,8,9,10,11,12,13]. The imbalance in testicular cytokines and growth factors may impair normal spermatogenesis [14].

Acute myeloid leukemia (AML) affected approximately one million people in 2015, resulting in 147,000 deaths worldwide; males are affected more frequently than females [15,16]. In 2019, the mortality of AML reached around 28% of all cases of leukemia [17]. Pediatric AML generally occurs de novo, with the incidence decreasing gradually with ages up to 9 years old and then gradually increasing into adulthood [18]. Different studies demonstrated the presence of spermatogonial cells in the testicular biopsies isolated from prepubertal AML patients before and after chemotherapy treatment [19,20,21]. Recently, we established a prepubertal AML mouse model and showed that AML induced clear damage on the histology of the seminiferous tubules (STs) and the cellular composition, mainly the meiotic/post-meiotic cells. We suggested that this effect could be related to the imbalance in testicular growth factors (SCF, GDNF, and MCSF) that are important in providing growth and/or differentiation factors and a normal niche/microenvironment, thus reducing the differentiation and development of the meiotic and post-meiotic cells [22]. In parallel, AML disrupted the balance between apoptosis and the proliferation processes in the STs [22]. Furthermore, we showed that AML led to an imbalance in testicular interleukin-6 (IL-6) (a pro-inflammatory cytokine) and IL-10 (an anti-inflammatory cytokine), which, in turn, may lead to testicular inflammation, which may impair normal spermatogenesis [22]. IL-10 and IL-6 are secreted by testicular non-immune cells, Sertoli, Leydig, and germ cells under normal conditions and significantly increased under pathological conditions [23,24]. Prepubertal AML patients are treated with cytarabine (CYT) and anthracycline drugs [25,26]. Our lab demonstrated that CYT impaired spermatogenesis and affected testicular development and testicular function by inducing DNA damage and apoptosis and reducing germ cell proliferation in adult- and juvenile-treated mice [22,27]. Furthermore, it decreased the sperm count in the treated group compared to the control group [22,27]. Additional studies showed that CYT-impaired spermatogenesis increased the percentage of tubules with apoptotic cells and decreased sperm parameters in adult mice [27,28,29]. 

The granulocyte colony-stimulating factor (GCSF) is a glycoprotein cytokine responsible for the proliferation and differentiation of bone marrow granulocyte progenitors into mature granulocytes [30,31]. It is produced mainly by macrophages, fibroblasts, and endothelial cells, and acts as a potent stimulus to both increase and accelerate neutrophil production [32,33,34,35]. According to previous studies, testicular macrophages, Sertoli, and Leydig cells produce GCSF, and its receptor is present in the SSCs [27,36]. Recently, our group demonstrated the presence of GCSF-R in sperm cells and that AML disease in adult mice did not affect the expression level of testicular GCSF compared to the control [27]. GCSF may act as a sensitizer of leukemic cells to the chemotherapy in AML patients; clinical studies show that the presence of GCSF in FLAG regimens increases the rate of disease-free survival of patients due to its effect of priming the leukemic cells to the chemotherapy [37,38,39,40,41]. Previous studies demonstrated that an injection of GCSF post-busulfan treatment improved spermatogenic recovery in the adult mouse model [42,43]. Our lab showed, for the first time, the administration of a pegylated form of GCSF into mice that had AML disease and treated with CYT restored the normal histology of the testis and epididymis and improved the functionality of the spermatogenic process, sperm parameters, and fertility capacity compared to the same treated groups without GCSF [27]. The FDA approved the first form of GCSF (Filgrastim) in 1991 as a treatment for neutropenia in cancer patients [44,45]. Recently, it was demonstrated that following busulfan chemotherapy, the GCSF treatment was associated with an improved spermatogenic recovery [42,43]. 

Our previous study examined the effect of GCSF administration on adult mice with AML disease and treated with CYT [27]. In the present study, we aim to evaluate the effect of GCSF administration on the development of spermatogenesis in adulthood of juvenile AML mice models treated with cytarabine.

## 2. Results 

### 2.1. Effect of GCSF on the Survival of Juvenile Mice Treated by AML Cells and CYT

**Figure 1:**
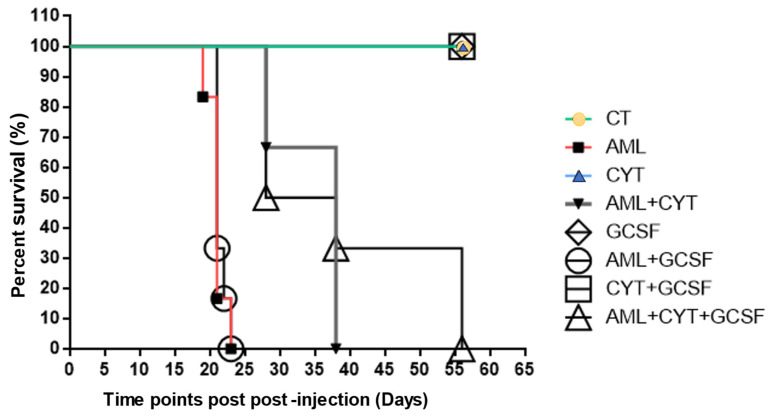
Effect of GCSF on the survival period of AML- and CYT-treated juvenile mice. Two-week-old mice divided into 8 groups; negative control without treatment (CT), i.p injected with AML cells (AML), i.p-injected with cytarabine (three times), injected with AML cells and cytarabine (AML+CYT), i.p-injected with GCSF (GCSF), i.p-injected with AML cells and GCSF (AML+GCSF), i.p-injected with AML cells and cytarabine and GCSF (AML+CYT+GCSF) (see Material and Methods section). Survival of the mice was followed up within 56 days. N (number of repeats of all treatments) = 4–7; *n* (number of mice examined in all the experiments for each treatment) = 9–24.

The survival of all treated groups was examined through 56 days post-injection. Our results showed that the injection of GCSF did not affect the survival of control CT, AML-, and CYT-treated juvenile mice. However, it extended the survival of the AML+CYT-injected juvenile group by 20 additional days (56 days) (Figure 1).

### 2.2. Effect of GCSF on the Testes Weight/Body Weight Ratio of AML- and CYT-Treated Juvenile Mice

**Figure 2:**
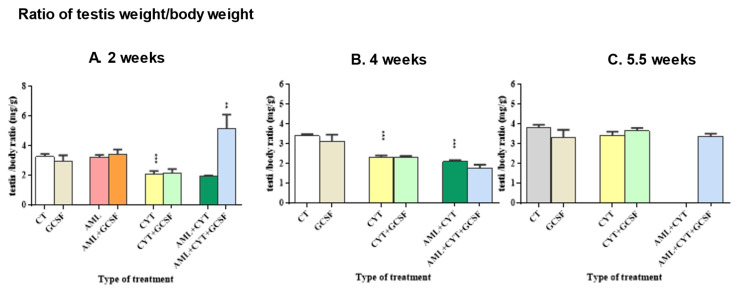
Effect of GCSF on the testes weight/body weight ratio of AML- and CYT-treated juvenile mice. Mice were treated as described in Figure 1. Testes weights (mg) and body weight (gram; g) were measured at several time points post-injection (2 weeks, **A**), 4 weeks (**B**), and 5.5 weeks (**C**). Ratio of testis weight/body weight is presented. N (number of repeats of all groups) = 4; *n* (number of mice examined in all the experiments for each group) = 2–13. Data are presented as mean ± SEM. ** (*p* < 0.01) and *** (*p* < 0.001) significant relative to the control group.

Injection of GCSF into juvenile mice (GCSF group) did not affect the testis weight/body weight ratio at the examined time points post-injection compared to the control group (CT) (Figure 2). Also, AML treatment did not affect the testis weight/body weight ratio 2 weeks post-injection compared to CT (Figure 2A). However, CYT-treated mice showed a significant decrease in the testis weight/body weight ratio 2 and 4 weeks post-injection compared to CT (Figure 2A,B), but it was similar to CT after 5.5 weeks post-injection. Mice treated with both AML and CYT (AML+CYT) showed a similar effect on the ratio of testis weight/body compared to CYT and a significant decrease compared to CT 2 and 4 weeks post-treatment (Figure 2A,B). However, injection of GCSF into AML- and CYT-treated mice (GCSF+AML+CYT) significantly increased the ratio of testis weight/body weight compared to AML+CYT group after 2 weeks of injection (Figure 2A) and was similar to CT groups after 5.5 weeks post-injection (Figure 2C) (the group of AML+CYT-treated mice did not survive the 5.5 weeks). Furthermore, the AML+CYT group that was treated with GCSF (GCSF+AML+CYT) showed a similar ratio of testis weight/body weight compared to the AML+CYT group after 4 weeks of treatment (Figure 2B). 

### 2.3. Effect of GCSF on the Testicular Histology of AML- and CYT-Treated Juvenile Mice

**Figure 3:**
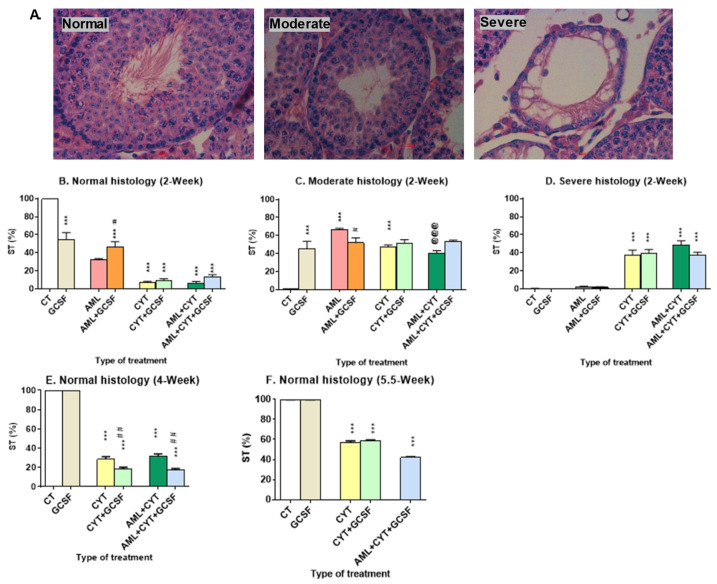
Effect of GCSF on seminiferous tubule (ST) histology of AML- and CYT-treated juvenile mice. Mice were treated as described in Figure 1. Histological sections from all treated groups (2 weeks post-injection) were stained with hematoxylin and eosin (**A**). The sections were classified according to histology of the seminiferous tubules into the following three classes: normal—seminiferous tubules with complete spermatogenesis (similar to tubules in the CT group (Normal), moderate—seminiferous tubules with partial damage, similar to tubules in AML group (Moderate), and seminiferous tubules with severe damage similar to CYT group (Severe). The percentages of tubules with the different classifications were calculated for the different treated groups and after different time points of treatments. The percentages of normal (**B**), moderate (**C**), and severe (**D**) histology were evaluated after 2 weeks. The normal histology for treatments after 4 weeks (**E**) and 5.5 weeks (**F**) is presented. N = 5–7; *n* = 9–13. *** (*p* < 0.001) significant relative to control group. @@@ (*p* < 0.001) significant relative to AML group. # (*p* < 0.05), ## (*p* < 0.01) significant in relative to the same group without GCSF. Scale bar: 500 px. Data are presented as mean ± SEM.

Testicular sections were collected from all study groups (CT, AML, CYT, and AM+CYT) with and without treatment with GCSF at several time points [depending on the survival of each treated group (2, 4, and 5.5 weeks)]. Sections were stained with hematoxylin and eosin for histological analyses (Figure 3A). The seminiferous tubules (STs) were analyzed according to the following classification scale that was previously mentioned in our previous study: normal—histology of STs similar to those in CT (tubule without damage), moderate—histology of STs similar to those in AML (tubule with fewer vacuoles), and severe—histology of STs similar to those in CYT (single cell layer, and much more vacuoles) [22]. The quantification results of this classification are summarized in (Figure 3B–F). Our results showed that 2 weeks post-injection of GCSF, the percentage of STs with normal histology significantly decreased, a significant increase in the percentage of STs with moderate histology and no effect on severe histology of the STs compared to control (CT) (Figure 3B–D). However, 4 weeks and 5.5 weeks post-injection of GCSF did not affect the histology of STs compared to CT (Figure 3E,F). On the other hand, 2 weeks post-injection of GCSF to AML-treated group significantly increase in the percentages of normal histology of the STs, but a significant decrease in the percentages of the moderate histology of the STs and without change in the severe histology of the STs compared to AML group. However, it was still significantly lower compared to the CT group (Figure 3B–D). There are no data available for the 4 and 5.5 weeks post-injection of GCSF and AML (AML+GCSF) since this group of mice did not survive that long.

An injection of GCSF into CYT-(alone)-, (CYT+GCSF)-, or into (CYT+AML)- (AML+CYT+GCSF)-treated mice did not affect the histology of STs compared to the relevant groups without GCSF 2 and 5.5 weeks post-treatment (Figure 3B–D,F). However, injection of GCSF into CYT (alone), (CYT+GCSF), or into (CYT+AML)-treated mice (AML+CYT+GCSF) significantly decreased the percentages of normal histology of their STs 4 weeks post-treatment (Figure 3F). The histology of the seminiferous tubules 4 weeks and 5.5 weeks post-treatment (Figure 3E and Figure 3F, respectively) was normal or moderate. 

### 2.4. Effect of GCSF on Apoptosis of Spermatogenic Cells in Testicular Tissue of CYT- and AML-Treated Juvenile Mice

**Figure 4:**
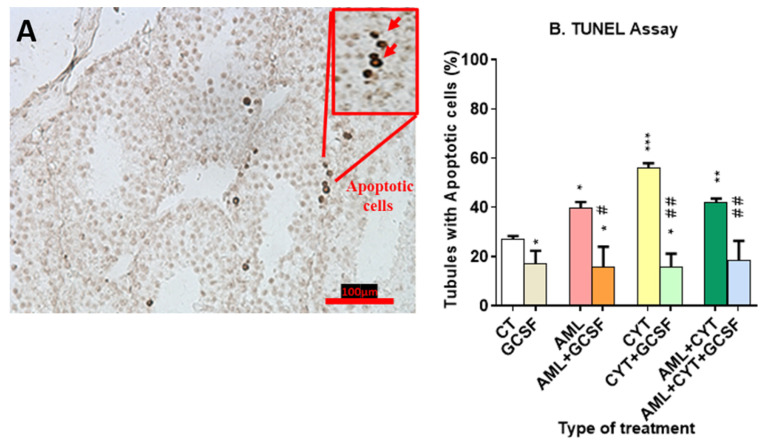
Effect of GCSF on apoptosis of spermatogenic cells in testicular tissue of AML- and CYT-treated juvenile mice. Mice were treated as described in Figure 1. Testes were removed 2 weeks post-injection and fixed for TUNEL assay. Fixed testicular tissues were examined by using TUNEL assay to identify apoptotic cells. Apoptosis is defined as tubules with apoptotic cells and presented as the % of tubules with apoptotic cells as mentioned in a previous study [27] (**A**). Quantification of the TUNEL assay staining for 2 weeks post-injection is presented (**B**). N (number of repeats of all treatments) = 3–7; *n* (number of mice examined from all the experiments for each treatment (TUNEL assay) = 6–7/group; number of tubules/group = 40–100. * (*p* < 0.05), ** (*p* < 0.01) *** (*p* < 0.001) significant relative to control group. # (*p* < 0.05), ## (*p* < 0.01) significant in relative to the same group without GCSF. Scale bar: 100 µm. Data are presented as mean ± SEM.

Apoptosis of cells in the testicular sections was examined using the tunel assay (Figure 4A). Juvenile mice treated with AML-alone, CYT-alone, or both AML and CYT (AML+CYT) showed a significant increase in the percentages of tubules with apoptotic cells (Figure 4B). On the other hand, the administration of GCSF into control mice (GCSF), or mice treated with AML-alone (AML+GCSF), CYT-alone (CYT+GCSF), or with combination of AML and CYT (AML+CYT+GCSF) significantly decreased the percentage of STs with apoptotic cells compared to the relevant groups without GCSF 2 weeks post-injection (Figure 4B). These results indicate that GCSF may have an anti-apoptotic effect on the testes of juvenile mice and reduce the apoptotic effects of the AML and CYT in testicular cells.

### 2.5. Effect of GCSF on the Expression Levels and the Number of Spermatogenic Cells in CYT- and AML-Treated Juvenile Mice

#### 2.5.1. Pre-Meiotic Stage

**Figure 5:**
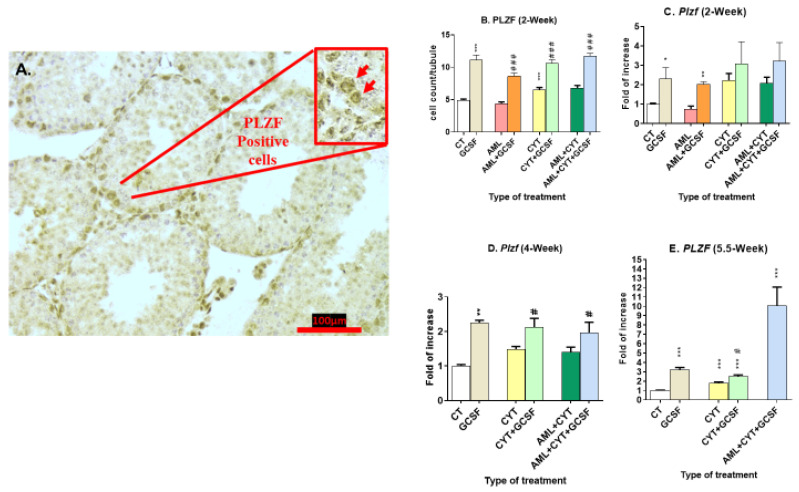
Effect of GCSF on the presence and expression levels of pre-meiotic marker (PLZF). Mice were treated as described in Figure 1. Testes were removed after 2, 4, or 5.5 weeks post-injection and fixed or used for RNA extraction. Fixed testicular tissues were immunohistochemically stained with specific anti-PLZF antibody to identify pre-meiotic cells (**A**). PLZF-positive stained cells are presented as PLZF count/tubule (**B**). The RNA expression levels of *Plzf* in the testes isolated from each treatment group in the different time points (2, 4, 5 weeks post-treatment) were examined by qPCR analysis using specific primers (**C**–**E**). The housekeeping gene (*Gapdh*) was used as internal control. The results are presented as fold of increase compared to *Gapdh*. Arrows indicate the location of PLZF-positive stained cells. N = 3–7; *n* = 5–7/group; * (*p* < 0.05), ** (*p* < 0.01), and *** (*p* < 0.001) significant relative to control group. # (*p* < 0.05), and ### (*p* < 0.001) significant in relative to the same group without GCSF. Scale bar: 100 µm. Data are presented as mean ± SEM.

Immunohistochemical staining (IHC) was used to examine the presence of PLZF in the testicular sections of mice after different weeks following the injection of GCSF into CYT- and AML-treated juvenile mice (Figure 5A, control mice). We examined at least 2–3 sections from each testis. We counted only round tubules. Our results showed that injection of GCSF into the normal group (GCSF) significantly increased the number of PLZF-stained cells/tubule compared to CT 2 weeks post-injection (Figure 5A). Also, the injection of GCSF into AML- (alone) (AML+GCSF), CYT-(alone) (CYT+GCSF), and AML+CYT (AML+CYT+GCSF) significantly increased the number of PLZF-stained cells/tubule compared to the relevant groups without GCSF 2 weeks post-injection (Figure 5A).

On the RNA expression levels, our results showed that the injection of GCSF-alone (GCSF) into juvenile mice significantly increased the RNA expression level of *Plzf* compared to CT 2, 4, and 5.5 weeks post-injection (Figure 5C–E). And the injection of GCSF into the AML-treated group (AML+GCSF) significantly increased the *Plzf* level compared to the AML-treated mice without GCSF 2 weeks post-injection (Figure 5B). We are not presenting data for 4 and 5.5 weeks post-injection of GCSF and AML (AML+GCSF) due to the fact that this group of mice died prior to these time points. Furthermore, the injection of GCSF into the CYT-alone (CYT+GCSF) or in combination with the AML-treated group (AML+CYT+GCSF) significantly increased the *Plzf* level compared to the relevant groups without GCSF 4 weeks and 5.5 weeks post (Figure 5D,E). However, the injection did not affect the *Plzf* level in those groups 2 weeks post-injection (Figure 5B).

#### 2.5.2. Meiotic/Post-Meiotic Stage 

**Figure 6:**
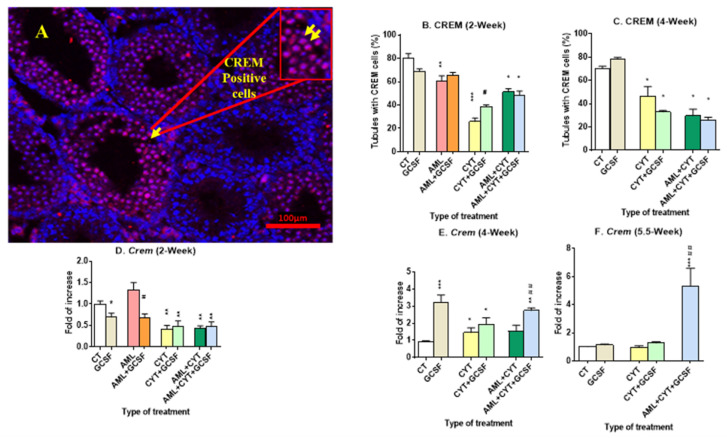
Effect of GCSF on the presence and expression levels of the meiotic marker (CREM). Mice were treated as described in Figure 1. Testes were removed after 2, 4, or 5.5 weeks and fixed or used for RNA extraction. Fixed testicular tissues were immune stained with specific anti-CREM antibody to identify meiotic cells (**A**). Tubules with more than 15 CREM-positive cells (**B**,**C**) are presented as % of tubules. The RNA expression levels of *Crem* (**D**–**F**) in the testes isolated from each treatment group were examined by qPCR analysis using specific primers, and housekeeping gene (*Gapdh*) was used as internal control. The results are presented as fold of increase compared to *Gapdh* (**D**–**F**). Arrows indicate the location of CREM-positive stained cells. N (number of repeats of all treatments) = 3–7; *n* = (number of mice examined from all the experiments for each treatment (immunostaining) = 5–7/group; number of tubules/group = 40–120; *n* = 5–7/group (RNA expression). * (*p* < 0.05), ** (*p* < 0.01), *** (*p* < 0.001) significant relative to control group. # (*p* < 0.05), ## (*p* < 0.01) significant in relative to the same group without GCSF. Scale bar: 100 µm. Data are presented as mean ± SEM.

**Figure 7:**
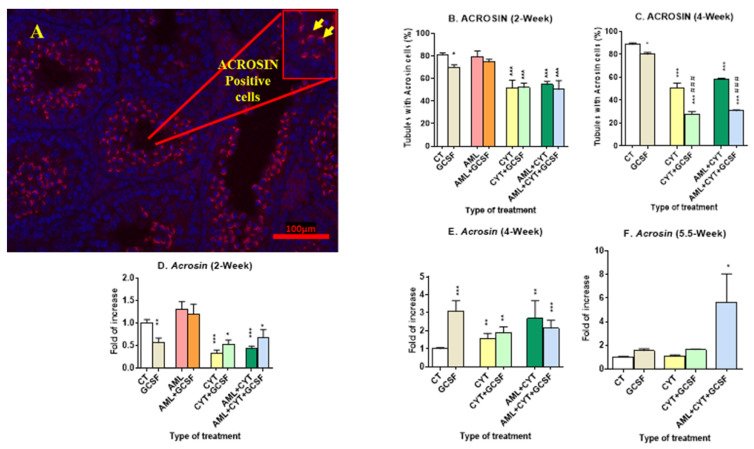
Effect of GCSF on the presence and expression levels of the meiotic/post-meiotic marker (ACROSIN). Mice were treated as described in Figure 1. Testes were removed after 2, 4, or 5.5 weeks and fixed or used for RNA extraction. Fixed testicular tissues were immune stained with specific anti-ACROSIN antibody to identify meiotic cells (**A**). Tubules with more than 15 ACROSIN-positive cells (**B**–**D**) are presented as % of tubules. The RNA expression levels of *Acrosin* (**D**–**F**) in the testes isolated from each treatment group were examined by qPCR analysis using specific primers, and housekeeping gene (*Gapdh*) was used as internal control. The results are presented as fold of increase compared to *Gapdh* (**B**–**D**). Arrows indicate the location of ACROSIN-positive stained cells. N (number of repeats of all treatments) = 3; *n* = (number of mice examined from all the experiments for each treatment (immunostaining) = 6–9/group; number of tubules/group = 40–120; *n* = 5–7/group (RNA expression). * (*p* < 0.05), ** (*p* < 0.01), *** (*p* < 0.001) significant relative to control group. ### (*p* < 0.001) significant in relative to the same group without GCSF. Scale bar: 100 µm. Data are presented as mean ± SEM.

We investigated the effect of GCSF on the presence (staining) of meiotic (CREM) (using immunofluorescence staining, Figure 6A; control mice) and post-meiotic (ACROSIN) (using immunofluorescence staining, Figure 7A; control mice) stages in testicular tissues from CYT- and AML-treated juvenile mice 2 and 4 weeks post-injection. We examined at least 2–3 sections from each testis. We counted only round tubules.

Our results showed that the injection of GCSF into the normal group (GCSF) did not affect the percentage of tubules with more than 15 CREM-stained cells, which we considered as a baseline. We scanned many sections of different treated groups before analysis, and we found that most of the tubules were stained with more than 15 positive cells of crem or/and acrosin; besides that, we found tubules without positive staining and tubules with less than 15 cells compared to CT 2 and 4 weeks post-injection (Figure 6B,C). On the RNA expression levels, GCSF significantly decreased the expression of *Crem* compared to CT 2 weeks post-injection (Figure 6D). While GCSF increased the expression of *Crem* 3-folds compared to CT 4 weeks post-injection (Figure 6E). 

On the other hand, the injection of GCSF into the AML-treated group (AML+GCSF) did not affect the percentage of tubules with more than 15 CREM-stained cells compared to AML-alone (AML) 2 weeks post-treatment (Figure 6B), while it decreased the RNA expression level of *Crem* in (AML+GCSF)-treated group compared to AML-alone (AML) 2 weeks post-treatment (Figure 6B). We are not presenting data for 4 weeks and 5.5 weeks post-injection of GCSF and AML (AML+GCSF) because this group of mice died prior to these time points.

An injection of GCSF into the CYT-alone-treated group (CYT+GCSF) significantly increased the percentage of tubules with more than 15 CREM-stained cells compared to CYT-alone (CYT) 2 weeks post-injection (Figure 6B). While GCSF did not affect the expression level of *Crem* in (CYT+GCSF) group compared to CYT-alone (CYT) 2, 4, and 5.5 weeks post-injection (Figure 6D–F). However, the injection of GCSF into the AML+CYT-treated group (AML+CYT+GCSF) significantly increased the RNA expression level of CREM compared to the AML+CYT-treated group without GCSF (AML+CYT) 4 weeks post-treatment (Figure 6E). On the other hand, GCSF did not affect the percentage of tubules with more than 15 CREM-stained cells in the same group 2 and 4 weeks post-injection (Figure 6B,C).

In parallel, our results show that injection of GCSF into the normal group (GCSF) significantly decreased the percentage of tubules with more than 15 ACROSIN-positive cells compared to CT 2 weeks and 4 weeks post-injection (Figure 7B,C). The RNA expression analysis confirms the results for 2 weeks post-injection (Figure 7D) but showed a 3-fold increase compared to CT of 4 weeks post-injection (Figure 7E). 

An injection of GCSF into the AML (AML+GCSF)-treated group did not affect the percentage of tubules with more than 15 ACROSIN-positive cells compared to CT 2 weeks post-injection (Figure 7B). However, the injection of GCSF into the CYT-alone (CYT+GCSF) or in combination with AML (AML+CYT+GCSF) significantly decreased the percentage of tubules with more than 15 ACROSIN-positive cells compared to the relevant groups without GCSF 4 weeks post-injection (Figure 7C). However, the injection of GCSF into the CYT-alone (CYT+GCSF) group or in combination with AML (AML+CYT+GCSF) did not affect the RNA expression level of *Acrosin* compared to the relevant groups without GCSF 2 and 4 weeks post-injection (Figure 7D,E).

### 2.6. Effect of GCSF on Sperm Parameters of Survived Treated Groups

#### 2.6.1. Sperm Concentration

**Figure 8:**
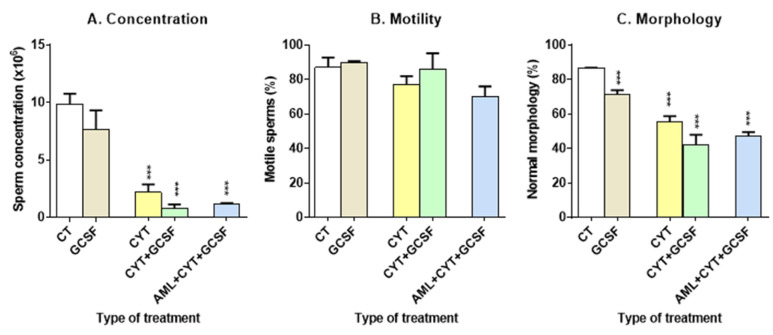

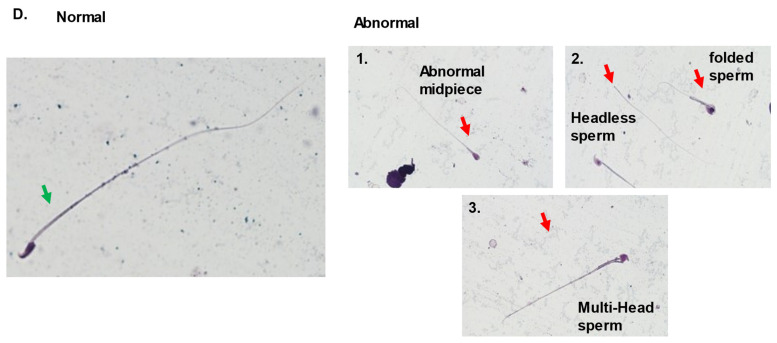
Effect of GCSF on sperm parameters of AML- and CYT-treated juvenile mice. Mice were treated as described in Figure 1. Sperm were extracted from the epididymis 5.5 weeks post-treatment. Sperm concentration was evaluated using a Makler counting chamber and determined according to WHO criteria (**A**). Sperm motility/immotility was evaluated using a Makler counting chamber and determined as a percentage of total sperm according to WHO criteria (**B**). Sperm morphology was evaluated following staining with Diff-Quick stain (**C**). Green arrow represents normal morphology, red arrows represent midpiece (1) and head defects (2 and 3) (**D**). N (number of repeats of all treatments) = 3; *n* = (number of mice examined from all the experiments for each treatment) = 2–5/group. For each mouse, we count 2 samples. *** (*p* < 0.001) significant relative to control group. Data are presented as mean ± SEM.

Here, we showed that the injection of GCSF into the normal group (GCSF) showed a similar concentration to CT 5.5 weeks post-injection (7.650 ± 1.639 × 10^6^/mL vs. 9.850 ± 0.8919 × 10^6^/mL) (Figure 8A). However, CYT-alone significantly decreased the sperm concentration compared to CT 5.5 weeks post-injection (2.168 ± 0.6871 × 10^6^/mL vs. 9.850 ± 0.8919 × 10^6^/mL) (Figure 8A), and the injection of GCSF to the CYT group (CYT+GCSF) did not affect the concentration of sperm compared to CYT without GCSF (CYT) 5.5 weeks post-injection (Figure 8A). Furthermore, the injection of GCSF into the AML+CYT group (AML+CYT+GCSF) significantly increased the sperm concentration from zero at 4 weeks post-injection, as mentioned in our previous study [22] to (1.200 ± 0.0400 × 10^6^/mL) 5.5 weeks post-injection (Figure 8A), but it was still significantly lower compared to the control group (CT) (1.200 ± 0.0400 × 10^6^/mL vs. 9.850 ± 0.8919 × 10^6^/mL) (Figure 8A). 

#### 2.6.2. Sperm Motility and Morphology 

Our results showed that the injection of GCSF into the CT group (GCSF) did not affect sperm motility after 5.5 weeks compared to CT (Figure 8B). In addition, an examination of sperm motility in groups that remained alive 5.5 weeks post-injection of GCSF (CYT+GCSF) and (CYT+AML+GCSF) showed no different effect compared to the control group (CT; GCSF) (Figure 8B). On the other hand, the evaluation of the effect of GCSF on the morphology of the sperm (Figure 8D) from the survival groups (CYT, CYT+GCSF, and AML+CYT+GCSF) (Figure 8C) showed that the percentage of the normal morphology of the sperms significantly decreased after all types of treatment compared to CT and that an injection of GCSF did not improve the normal morphology of sperms in the treated groups (Figure 8C). 

### 2.7. Effect of GCSF on the Levels of Testicular Pro-inflammatory and Anti-Inflammatory Cytokines of CYT- and AML-Treated Juvenile Mice

**Figure 9:**
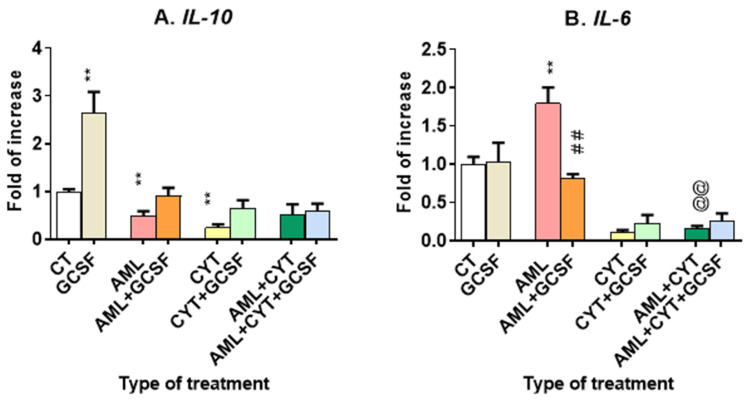
Effect of GCSF on the expression levels of testicular *IL-6* and *IL-10* in AML- and CYT-treated juvenile mice. Mice were treated as described in Figure 1. Testes were removed after 2 weeks and used for RNA extraction. The RNA expression levels of *IL-10* (**A**) and *IL-6* (**B**) in the testes isolated from each treatment group were examined by qPCR analysis using specific primers, and housekeeping gene (*Gapdh*) was used as internal control. The results are presented as fold of increase compared to Gapdh. N (Number of repeats) = 4; *n* (number of samples)= 5–7/group (RNA expression). ** (*p* < 0.01) significant relative to control group. @@ (*p* < 0.01) significant relative to AML group. ## (*p* < 0.01) significant in relative to the same group without GCSF. Data are presented as mean ± SEM.

Here we examined the possible effect of GCSF on the expression levels of testicular *Il-10* and *Il-6* in CYT- and AML-treated juvenile mice 2 weeks post-injection. Our results showed that the injection of GCSF into the CT group (GCSF) significantly increased the RNA expression level of the anti-inflammatory cytokine (*Il-10*) compared to CT 2 weeks post-injection (Figure 9A), but it did not affect the RNA expression level of the pro-inflammatory cytokine (*Il-6*) at the same time point post-treatment (Figure 9B). The injection of GCSF into the AML-treated group (AML+GCSF) reversed the abnormal levels of (*Il-10* and *Il-6*) to normal levels compared to CT 2 weeks post-treatment (Figure 9A,B). However, the injection of GCSF to the CYT-alone (CYT+GCSF) or in combination with AML (AML+CYT+GCSF) did not affect the RNA expression level of (*Il-10* and *Il-6*) compared to the relevant groups without GCSF 2 weeks post-injection (Figure 9A,B).

## 3. Discussion 

In the present study, we demonstrated the protective effect of GCSF administration on juvenile AML mice models treated with cytarabine on the development of spermatogenesis in their adulthood age. 

According to studies in our and other labs, testicular macrophages, and Leydig cells produce GCSF and express its receptor on the SSCs under normal conditions [27,36]. 

The injection of GCSF following the AML disease did not affect the survival of the treated mice. However, the injection of GCSF after AML and CYT treatment improved mice survival by a mean of 20 additional days compared to AML+CYT-treated mice without GCSF (from 36 days to 56 days). 

The administration of GCSF into all types of treatments of juvenile mice did not affect the testes’ weight at all examined time points post-injection. These, in contrast to our recent study results using adult AML-model, those results showed a positive effect of GCSF on testes weight [27]. Our results of testicular weight (and ratio of testis weight/body weight) are in correlation with the histological analysis of the seminiferous tubules, where we did not find a significant effect of GCSF on the normal seminiferous tubule histology from AML-, CYT-, or (AML+CYT)-treated groups after 2 and 5.5 weeks post-injection compared to the GCSF control group or the relevant treated group. However, after 4 weeks, the GCSF injection impaired the normal histology and increased the moderate histology compared to the relevant control. These results may suggest different mechanisms of regulation of GCSF on the adult testis and during its development. Furthermore, comparing the percentages of normal STs of the AML+CYT+GCSF-treated groups 4 weeks and 5.5 weeks post-injection shows an increase of 17%–42%, which may suggest that the positive effect of GCSF on the histology of the STs needs more time to manifest when added to the CYT regimen of treatment. Additionally, we showed that the injection of GCSF into the normal group or the AML (alone), CYT (alone), or in combination with AML significantly decreased the percentage of STs with apoptotic cells compared to the relevant groups without GCSF and CT only at 2 weeks post-treatment. These results may suggest that GCSF decreases apoptosis under severe conditions. It is possible that the decrease in the percentage of tubules with apoptotic cells could be related to the increase in normal seminiferous tubules following AML disease. This finding is in harmony with other reports that demonstrated that a GCSF injection prevented testicular damage from chemotherapy and radiotherapy by [27,36,42,46,47,48]. The anti-apoptotic activity of GCSF may be mediated via the down-regulation of the P-JNK and P-c-jun pathways and the up-regulation of STAT transcription factors [49,50].

We also examined the possible involvement of GCSF in protecting/restoring the spermatogenesis process. Here, we showed that the injection of GCSF into the normal group increased the examined SSCs markers (PLZF) 2 weeks post-injection. GCSF did not affect the number of the examined meiotic cells (CREM positive cells) and decreased the post-meiotic cells (ACROSIN positive cells) 4 weeks post-injection. Additionally, the GCSF-treated group showed similar results on the histology of STs, sperm concentration, and fertility capacity 5.5 weeks post-injection compared to the CT group. The injection of GCSF into the AML-treated group increased the SSCs markers but did not affect the meiotic/post-meiotic cells compared to the AML-treated group without an injection of GCSF 2 weeks post-injection. The injection of GCSF into the CYT group increased the SSCs and meiotic cells 2 weeks post-injection. In contrast, it decreased the post-meiotic cells 4 weeks post-treatment and did not affect the sperm concentration compared to the CYT-treated mice without an injection of GCSF. The injection of GCSF into the AML+CYT group increased the SSCs, while it did not affect the meiotic cells 4 weeks post-injection. In contrast, it decreased the post-meiotic cells 4 weeks post-injection. It is important to note that GCSF not only extended the survival of the AML+CYT-treated group by an additional 2 weeks, but it may have also increased the potential (at least according to the RNA results) of the future development of meiotic and/or post-meiotic stages. Furthermore, it is important to highlight that the administration of GCSF significantly increased the examined marker for SSCs (PLZF) (counts and/or RNA expression) compared to the relevant control groups. These results might be important in the field of male fertility preservation since the presence of these SSCs could be used in the future using the different technologies already proven in animal models (not yet in humans) to generate fertile sperm and even offspring [51]. Our results are in agreement with our recent study that showed a positive effect of GCSF administration on the spermatogenesis process of adult AML mice models [27]. Further support came from another group that examined the effect of GCSF on spermatogenic regeneration from surviving spermatogonia after busulfan chemotherapy and showed that mice treated with GCSF before or after busulfan treatment increased their numbers of testicular PLZF cells [42].

In addition, in this study, we demonstrated that the injection of GCSF into the normal group significantly increased the RNA expression level of *IL-10* but not *IL-6* compared to CT 2 weeks post-injection. GCSF did not affect the RNA expression level of both *IL-10* and *IL-6* when injected into CYT-alone or in combination with AML compared to the relevant groups without GCSF 2 weeks post-injection. However, the injection of GCSF into the AML-treated group reversed the expression of both *IL-10* and *IL-6* to normal levels compared to CT 2 weeks post-injection. We suggest that GCSF has an anti-inflammatory effect in the testis under normal conditions by increasing *IL-10* levels, but under pathological conditions (AML) by decreasing the inflammatory cytokine *IL-6*. 

We found that administration of GCSF into the AML+CYT-treated mice significantly increased sperm concentration from zero at 4 weeks post-injection, as mentioned in our previous study [22], to (1.2 ± 0.04 × 10^6^/mL) at 5.5 weeks post-injection. There was no significant effect of GCSF on sperm motility and morphology following the treatment of CYT. However, the percentage of motility (was similar to the control) and morphology (was lower compared to the control) of the sperm were high following the GCSF treatment of the AML+CYT group (AML+CYT+GCSF) compared to (AML+CYT) group. These findings may suggest that GCSF may partially protect against AML and cytarabine testicular damage and induce sperm production. On the other hand, the amount of the produced sperm was not sufficient for natural fertility (as examined by mating) (the data are not shown). These results are encouraging because the presence of this amount of sperm could be used in assisted reproduction technologies, such as in vitro fertilization or intra-cytoplasmic sperm injection. 

In conclusion, our results show that AML-alone, in the male mouse at prepubertal age, affects their fertility in the adult age. In addition, the treatment of AML-treated prepubertal mice with CYT could adversely affect their fertility at adult age. The addition of GCSF to the regimen of AML after CYT may enhance the microenvironment that surrounds the germ cells and thus may improve the development of spermatogenesis and generation of sperm that could be used in assisted reproduction technologies to fertilize oocytes. Thus, our study may assist in the development of future therapeutic strategies for male fertility preservation of cancer patients, mainly AML-treated prepubertal cancer patients treated with chemotherapy. 

## 4. Materials and Methods 

### 4.1. Animals

C57Bl/6 mice (14-day-old) were purchased from Harlen Laboratories Ltd., Jerusalem, Israel. They were appropriately handled at the animal house in the Faculty of Health Science at Ben-Gurion University of the Negev, Israel, according to the approved handling protocol. Mice were sacrificed at several time points. 

This study was performed under the Guiding Principles for the Care and Use of Research Animals disseminated by the Society for the Study of Reproduction and was confirmed by the Ben-Gurion University Ethics Committee for Animal Use in Research (IL-93-21-2020). 

### 4.2. Preparations of the AML Cell Line, Cytarabine, and GCSF for Injection

**Scheme 1:**
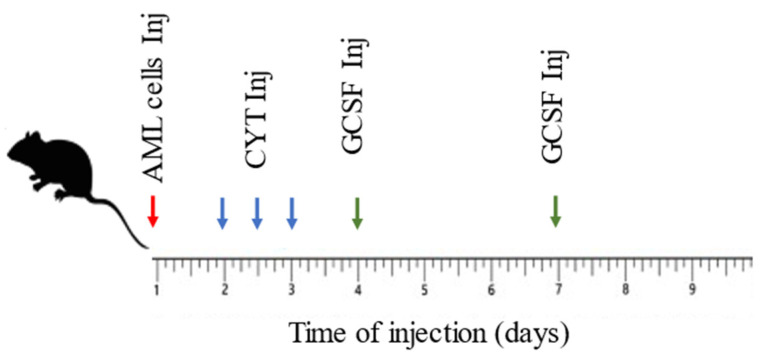
Experimental flow scheme. Red arrow represents the injection of the AML cells, blue arrows represent the injection of the CYT injection (3-times every 12 h), and green arrows represent the injection of the GCSF injection (2-times every 72 h).

The preparation and the intraperitoneal injection (ip) of the mouse AML cell line (murine C1498) (3 × 10^4^ cells/100 µL) was performed according to our previous study [22]. The powder of cytarabine was purchased from SIGMA (Sigma-Aldrich Israel Ltd., Rehovot, Israel) and prepared for injection (ip) according to our previous study [22]. Cytarabine (140 mg/kg) was injected (i.p) into each premature mouse. The injections were performed every 12 h, three times after 24 h of C1498 cells injection. The GCSF was purchased from (Amgen New Zealand Limited., Auckland, New Zealand) and was diluted in sterile PBS. GCSF (30 µg/kg/dose/100 μL) was injected (ip) into each mouse. The injections were performed every three days, two times after 24 h of the last CYT injection (Scheme 1).

### 4.3. Mouse Survival 

The survival of mice was examined every day for 56 days.

### 4.4. Testes Weight and Histological Analysis

Mice were sacrificed by using CO_2_, and testes were removed from each mouse that was involved in the study and weighed at different time points post-injection (2.4, and 5.5 weeks). Testes were fixed in Bouin’s solution (Kaltek, Italy) and embedded in paraffin, as mentioned in the previous study [22]. Hematoxylin and eosin staining (Pioneer Research Chemicals, Germany) was performed, and the results were analyzed according to a previous study [22]. 

### 4.5. Evaluation of Sperm Parameters

Mice were sacrificed 5.5 weeks post-injection, and the epididymis was removed; sperm cells were extracted from the epididymis by squeezing in a Petri dish plate. Cells were collected in a small tube. Collected sperm were examined for concentration using a Makler counting chamber. In total, 10 µL of each sample was transferred to a chamber, and cells were counted at a total microscope magnification of ×400. For sperm motility evaluation, only motile cells were counted without differentiation of motility type. Then semen smears were made and stained using Quik Stain (Biological Industries, Cromwell, CT, USA). Evaluation of sperm morphology was performed using an upright microscope under magnification with an objective lens at ×100 (using immersion oil). This was performed according to WHO criteria [52]. 

### 4.6. TUNEL Immunohistochemistry

Immunohistochemical apoptotic detection was carried out using a commercial kit (DeadEnd™ Fluorometric TUNEL System, Promega, Madison, WI, USA). The assay was conducted according to the manufacturer’s instructions. And the analysis of the results was performed according to a previous study [22]. 

### 4.7. Immunofluorescence Staining (IF)

Deparaffinization and dehydration procedures were carried out before staining. IF was performed according to a previous study [22].

The primary antibody was diluted with the blocking solution according to the instructions of the manufacturer. Each slide included a negative control (NC) section that was not incubated with the primary antibody. For IF staining of ACROSIN, primary antibodies (Polyclonal rabbit anti-mouse, 1:1000; Novus Biologicals, LLC, Centennial, CO, USA, Cat. No. A113694) were used. For IF staining of CREM, primary antibodies (Polyclonal rabbit anti-mouse, 1:200; Novus, Cambridge, UK, Cat. No. NBP2-1600) were used.

Secondary antibodies used in this study were the following: donkey anti-rabbit IgG (Cy3) (Jackson immune research, West Grove, PA, USA; 1:700). The slides were examined for staining using a fluorescence microscope (Nikon Eclipse 50 I; Tokyo, Japan). And the slides were allowed to dry and were then ready for analysis by using a fluorescence microscope (Nikon Eclipse 50 I; Tokyo, Japan). [22]. 

### 4.8. Immunohistochemistry Staining of Testicular Tissues

Immunohistochemistry staining was performed as described in the manufacturer’s protocol. The slides were prepared exactly as mentioned in immunofluorescent staining. The primary antibodies were used as follows: PLZF (Polyclonal rabbit anti-mouse, 1:100, Santa Cruz Biotechnology, Santa Cruz, CA, USA, Cat. No. sc-22839). After overnight incubation at 4 °C, the slides were washed, and the specific secondary antibodies were added compatibly to the primary antibodies. The specificity of the staining was also examined in the testicular tissue using the relevant IgG isotype as the negative control. The slides were examined for staining using a fluorescence microscope (Nikon Eclipse 50 I; Tokyo, Japan) [22].

### 4.9. Real-Time Quantitative PCR

RNA was extracted according to the manufacturer protocol (Sigma (GenElute Mannalian Total RNA Miniprep)) and according to a previous study [53]. cDNA synthesis was performed according to the qScript cDNA Synthesis Kit (Quantabio, Beverly, MA, USA) using random hexamers, and qPCR was performed using specific primers for each of the following examined marker: *Gapdh* Fw-5-5′ACCACAGTCCATGCCATCAC-3′, Rv-5′-CACCACCCTGTTGCTGTAGCC-3′. *Plzf* Fw-5′-AGCTTGAAATACGTGGCCAGA-3′, Rw-5′-TGAGCAGTTCACACTTCATCCC-3′. *Crem* Fw-5′-TTCTTTCACGAAGACCCTCA-3′, Rw-5′-TGTTAGGTGGTGTCCTTCT-3′. *Acrosin* Fw-5′-TGTCCGTGGTTGCCAAGGATAACA-3′, Rv-5′-AATCCGGGTACCTGCTTGTGAGTT-3′. *IL-10* Fw-5′- CGGGAAGACAATAACTGCACCC-3′, Rv-5′- CGGTTAGCAGTATGTTGTCCAGC-3′.

*IL-6* Fw-5′-GACGATACCACTCCCAACAGACC-3′, Rv-5′-ATGCTTAGGCATAACGCACTAGGTT-3′.

The qPCR reaction was performed and analyzed as described in a previous study [54]. The relative quantity of gene expression was analyzed using the 2−ΔΔCt method. The results are expressed as the fold of increase related to the *Gapdh* of the same examined sample.

### 4.10. Statistical Analysis

The statistics values were calculated according to Av. ± SEM. The statistical significance was examined by t-test or Mann–Whitney test and was shown as *p*-value: *, @, $ *p* < 0.05, **, @@, $$ *p*-value < 0.01, ***, @@@, $$$ *p*-value < 0.001.

## Data Availability

The data that support the findings of this study are available from the corresponding author upon reasonable request.
